# Cell-Free Circulating Plasma hTERT mRNA Is a Useful Marker for Prostate Cancer Diagnosis and Is Associated with Poor Prognosis Tumor Characteristics

**DOI:** 10.1371/journal.pone.0043470

**Published:** 2012-08-20

**Authors:** José A. March-Villalba, José M. Martínez-Jabaloyas, María J. Herrero, Jose Santamaria, Salvador F. Aliño, Francisco Dasí

**Affiliations:** 1 Fundación Investigación Hospital Clínico Universitario de Valencia, Instituto de Investigación INCLIVA, Valencia, Spain; 2 Urology Unite, Hospital Clínico Universitario de Valencia, Valencia, Spain; 3 Department of Pharmacology, School of Medicine, University of Valencia, Valencia, Spain; 4 Department of Physiology, School of Medicine, University of Valencia, Valencia, Spain; IPO, Inst Port Oncology, Portugal

## Abstract

**Background:**

Serum prostate-specific antigen (PSA) is the most widely used marker for diagnosing prostate cancer (PCa). It lacks specificity and predictive value, resulting in inaccurate diagnoses and overtreatment of the disease. The aim of this study was to assess the usefulness of plasma telomerase reverse transcriptase (hTERT) mRNA as a diagnostic and prognostic tool for PCa and its association with clinicopathological parameters of tumors.

**Principal Findings:**

Plasma hTERT mRNA levels were determined by qRT-PCR in 105 consecutive patients with elevated PSA levels and in 68 healthy volunteers. The diagnostic accuracy, the efficacy as a prognostic factor of biochemical recurrence and the association with tumor clinicopathological parameters of plasma hTERT mRNA and serum PSA tests were determined using univariate and multivariate analyses. The results show that plasma hTERT mRNA is a non-invasive biomarker for PCa diagnosis that shows higher sensitivity (85% vs. 83%), specificity (90% vs. 47%), positive predictive value (83% vs. 56%), and negative predictive value (92% vs. 77%) than serum PSA. Plasma hTERT mRNA is significantly associated with poor prognosis tumor clinicopathological parameters and is a significant independent predictor of PCa (p<0.0001). Univariate analysis identified plasma hTERT mRNA (but not serum PSA) as a significant prognostic factor of biochemical recurrence. Plasma hTERT mRNA Kaplan-Meier curves confirmed the significant differences between groups and patients with higher levels than the cut-off value showed diminished recurrence-free survival (p = 0.004), whereas no differences were observed with serum PSA (p = 0.38). Multivariate analysis indicated that plasma hTERT mRNA (but not serum PSA) and stage were significantly associated with biochemical recurrence.

**Conclusions:**

Overall, these findings indicate that hTERT mRNA is a useful non-invasive tumor marker for the molecular diagnosis of PCa, affording a greater diagnostic and prognostic accuracy than the PSA assay and may be of relevance in the follow-up of the disease.

## Introduction

Prostate cancer (PCa) is, with the exception of skin cancer, the most frequently diagnosed cancer among men in developed societies and the second cause of death from cancer after lung cancer [1]. Diagnosis of PCa has increased dramatically over the last decade, thanks largely to the extensive use of serum prostate specific antigen (PSA) tests [2]. When serum PSA rises above the threshold of 2.5–4.0 ng/ml, the patient is often considered a candidate for a biopsy in order to rule out the risk of cancer. However, the use of serum PSA to screen for PCa is controversial and not unanimously recommended by the international medical community. This discrepancy is due to the fact that the PSA test has limited specificity and predictive value, resulting in a significant number of false positives and, consequently, unnecessary biopsies [3].

There are several reasons why it is difficult to interpret the PSA test and to make an accurate decision about whether or not to perform a biopsy. First, up to 15% of cancer cases (some of them high-grade PCa) are not diagnosed in men with PSA levels below the cut-off level [4]. Second, although high serum levels of PSA are associated with PCa, they can also indicate other much less serious conditions, such as acute bacterial prostatitis, benign prostatic hyperplasia (BPH), cystitis, ejaculation, perineal trauma or recent surgical procedures in the urinary tract [2]. Data from the European Randomized Study of Screening for Prostate Cancer (ERSPC) showed that from 10–20% of men had abnormal PSA levels and therefore fulfilled the current criterion for a biopsy, though only around 24% of the men in question eventually developed PCa [5].

Moreover, the results of randomized clinical trials have demonstrated that PSA screening produces inconsistent results and has only a limited benefit with respect to PCa mortality. The ERSPC and the Göteborg studies revealed a moderately reduced PCa mortality rate [5], [6], whereas the Prostate, Lung, Colorectal, and Ovarian Cancer Screening (PLCO) trial showed no decrease in said rate [7]. Although high PSA levels are often associated with a more aggressive progression of PCa, they do not always correlate with the biological behavior of the disease, thereby leading to overdiagnosis and overtreatment, with the unnecessary risks of urinary, sexual and bowel dysfunction that this entails [8].

In this context, new biomarkers are radically needed to detect clinically relevant PCa and perform early and accurate diagnoses of the disease, thus reducing the number of biopsies while successfully detecting as many cases of PCa as possible.

To this end, human telomerase is one of the most promising tumor markers currently under investigation. Telomerase activity has been found to be elevated in 85–100% of cancer patients, whereas it is low or undetectable in normal somatic cells [9]. Several researchers have reported the detection of telomerase reverse transcriptase (hTERT) mRNA in plasma/serum of several types of cancers, including PCa [10]–[17]. In a previous study of a small number of patients, our group detected hTERT mRNA in the plasma of PCa patients and observed that its quantification constituted a sensitive and specific method for identifying PCa patients. Moreover, when considered in combination with PSA, it was an effective marker for early PCa diagnosis [16].

However, none of these studies have compared the presence of hTERT mRNA in plasma/serum of PCa patients with the clinicopathological characteristics of tumors. Therefore, the aim of the present study was to: i) study, in a large population of patients, the clinical relevance of hTERT plasma mRNA as a marker of PCa and, ii) analyze the distribution of clinicopathological parameters with respect to hTERT mRNA plasma levels in PCa patients.

## Materials and Methods

### Patients and Sample Collection

One hundred and five patients who were admitted consecutively to the Hospital Clínico Universitario Valencia (HCUV) were included in the study. Eligibility criteria were as follows: (1) patients with elevated serum PSA levels (PSA cut-off point ≥4.0 ng/ml), (2) no prior diagnosis of cancer of any type; and (3) no treatment for PCa prior to blood collection.

All samples were analyzed prospectively with no knowledge of subjects’ clinicopathological status. Patients were classified according to histopathological criteria and assigned to one of three groups: PCa (mean age±SD: 65.8±7.30 years, range: 49–80), prostatitis (mean age±SD: 65.9±5.69 years, range: 54–80), and BPH (mean age±SD: 62.4±8.45 years, range: 50–71) (Table 1). Patients with PCa were treated with radical prostatectomy (26/46; 57%), brachytherapy (3/46; 6%), radiotherapy (13/46; 28%) and androgen deprivation therapy (4/46; 9%). Median follow-up duration was 10.0 years (range: 9.5–10.4). Biochemical recurrence criteria are as indicated by the European Association of Urology guides; after radical retropubic prostatectomy, two consecutive PSA values of 0.2 ng/ml or greater and after radiation therapy, a rising PSA level >2 ng/ml above the nadir PSA [18]. According to these specific criteria, 7 patients had biochemical recurrence.

**Table 1:** Age, preoperative serum PSA and hTERT mRNA levels of controls and patients according to histopathological findings.

Group	Age (yr)*	PSA (ng/ml)**	hTERT mRNA**
Controls (n = 68)	61.0±6.32 (50–76)	1.3 (0.5–3.4)	0.07 (0.02–0.10)
BPH (n = 12)	62.4±8.45 (50–71)	6.9 (6.3–9.4)	0.03 (0.01–0.13)
Prostatitis (n = 47)	65.9±5.69 (54–80)	8 (5.7–11)	0.13 (0.03–0.31)
PCa (n = 46)	65.8±7.30 (49–80)	9.6 (7.5–13.4)	1.6 (0.7–4.08)

* Mean ±SD (Range).

** Median (Interquartile range).

Sixty-eight age-matched (mean age±SD: 61.0±6.32 years, range: 50–76) healthy volunteers with no symptoms in the lower urinary tract or history of PCa or any other type of cancer served as controls.

Peripheral venous blood samples were obtained from patients (prior to transrectal ultrasound-guided prostate biopsy) and healthy volunteers in 8-ml blood collection tubes containing sodium citrate gel and a density gradient medium (Vacutainer CPT 362761; Beckton Dickinson). Blood mononuclear cells and plasma were separated by centrifugation according to the manufacturer’s instructions.

This study was approved by the research ethics committee of the HCUV, and all participating subjects gave written informed consent prior to their inclusion in the study.

### RNA Isolation and Plasma hTERT mRNA Quantification

RNA was isolated from 250 µl of plasma with DNase I treatment, as previously described [11], [16]. cDNA synthesis and quantitative real-time PCR (qPCR) have been reported previously [16]. In short, purified RNA (200 ng) was reverse transcribed with random hexamers using the TaqMan Reverse Transcription reagents kit (Applied Biosystems) according to the manufacturer’s instructions. Five µl of the RT reaction were mixed with 45 µl of TaqMan Universal PCR Master Mix (Applied Biosystems) containing 250 nM of the forward and reverse primers and 125 nM of the TaqMan probe. Primers and TaqMan probes for hTERT and 18S rRNA and PCR conditions have been described elsewhere [17]. Plasma hTERT mRNA was normalized against plasma 18S rRNA mRNA. A standard curve was generated using the HT-29 colon carcinoma cell line (ATCC HTB-38). The cycle threshold (Ct) of each sample was measured using the ABI 7700 sequence detection system and transformed to ng of HT-29 hTERT and HT-29 18S rRNA using the standard curves generated in the same experiment. The ratio between ng of hTERT and ng of 18S rRNA represents the normalized hTERT for each sample.

Preoperative serum PSA values were measured by means of the standard technique employed in the HCUV (Elecsys total PSA assay; Roche Diagnostics).

### Clinicopathological Parameters

When the results of a PSA test pointed towards PCa, the patient underwent an ultrasound-guided prostate biopsy. The results of all biopsies were reviewed by experienced uro-pathologists and, if considered positive, the patient in question was treated according to the standard procedure followed in the Urology Unit at HCUV in cases of PCa. Tumor prognostic factors such as Gleason score, percent of positive prostate biopsies (calculated as the number of positive biopsies divided by the total number of biopsies performed), perineural invasion and lymphovascular invasion were determined. Clinical staging was performed by digital rectal examination (DRE), transrectal ultrasonography (TRUS) and magnetic resonance imaging.

### Statistical Methods

Plasma hTERT mRNA levels and serum PSA levels were compared with clinicopathological parameters using Mann-Whitney U and Kruskal-Wallis non-parametric tests, since a Kolmogorov-Smirnov test revealed that neither hTERT mRNA nor serum PSA was normally distributed. The accuracy of serum PSA and plasma hTERT mRNA assays in diagnosing PCa was determined by Receiver Operating Characteristic (ROC), the corresponding area under the curve (AUC ROC) and likelihood ratios (LR). The sensitivity and specificity of hTERT mRNA and preoperative serum PSA were calculated using the best cut-off value of the ROC curve, which was determined according to the highest point on the vertical axis and the furthest to the left on the horizontal axis. The association between plasma hTERT mRNA, serum PSA and clinicopathological parameters was analyzed using univariate analysis. Logistic regression was performed to determine significant independent pretreatment predictors of PCa diagnosis. The efficacy of plasma hTERT mRNA and serum PSA as predictors of biochemical recurrence was assessed by univariate and multivariate analyses. The odds ratios (ORs) computed by logistic regression and 95% confidence intervals (95% CI) were reported. All calculated p-values were two-tailed, and probability values of less than 0.05 were considered to be statistically significant.

## Results

### Plasma hTERT mRNA is a Useful Non-invasive Biomarker for PCa Diagnosis

Plasma hTERT mRNA levels were measured in 105 consecutive patients with elevated PSA levels (≥4.0 ng/ml) and 68 healthy volunteers. Patient age, preoperative serum PSA and plasma hTERT mRNA levels are shown in Table 1. No differences in age were observed between the different groups (ANOVA; p = 0.350).

**Figure 1:**
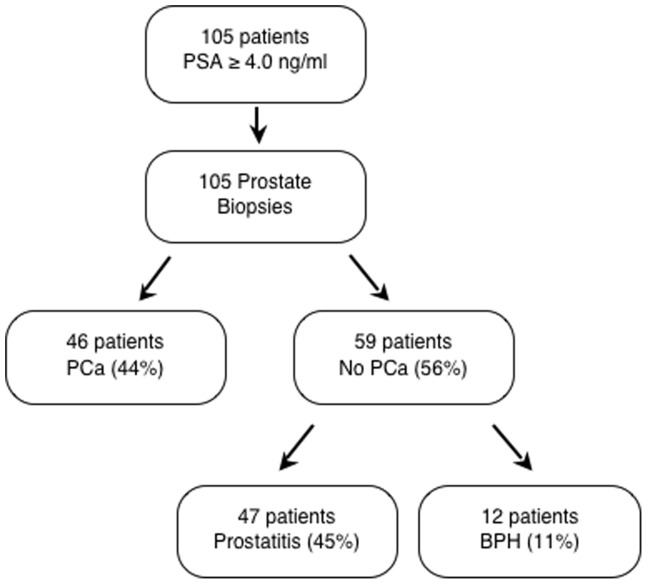
Flow diagram showing that PSA screening results in a high percentage of unnecessary biopsies. One hundred and five consecutive patients with elevated PSA levels underwent prostate biopsy. Forty-six of the 105 patients (44%) were diagnosed with PCa, and fifty-nine patients (56%) showed no evidence of cancer. PCa: Prostate Cancer BPH: Benign Prostatic Hyperplasia.

**Figure 2:**
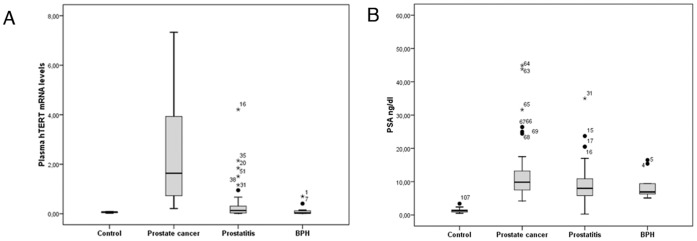
Boxplot diagram showing hTERT mRNA, serum PSA levels and histopathological findings. Data are expressed as median and interquartile range. A) Quantification of plasma hTERT mRNA levels discriminates between PCa patients and healthy individuals (p<0.001). B) Although PSA levels were significantly higher in PCa patients than in controls (p<0.001), quantification of serum PSA was unable to discriminate between PCa, BPH and prostatitis patients. BPH = Benign Prostatic Hyperplasia.

PCa screening using PSA as the only indicator for prostate biopsy revealed that forty-six of the 105 patients were diagnosed with PCa (43.8%), 47 had prostatitis (44.8%) and 12 suffered from BPH (11.4%) (Figure 1). Distribution of plasma hTERT mRNA levels in patients with PCa, prostatitis or BPH and in controls is shown in Figure 2A. Plasma hTERT mRNA median values were significantly higher (p<0.001) among the PCa patients (median; interquartile range: 1.6; 0.7–4.08) than in the control (0.07; 0.02–0.10), prostatitis (0.13; 0.03–0.31) and BPH (0.03; 0.01–0.13) groups. Preoperative serum PSA was significantly higher (p<0.001) in patients than among controls, although no significant differences were detected between the PCa group and the prostatitis or BPH groups (Figure 2B).

All patients and control samples showed 18S rRNA amplification indicating the integrity of the isolated RNA (data not shown).

### Plasma hTERT mRNA Shows Higher Diagnostic Accuracy than Serum PSA in PCa Diagnosis

**Figure 3:**
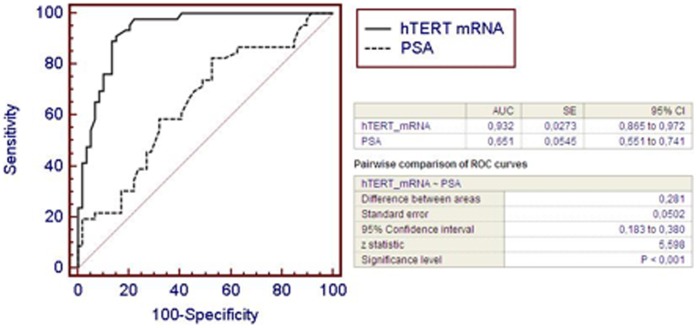
hTERT mRNA diagnostic accuracy. Comparison with serum PSA. ROC curves show that plasma hTERT mRNA, but not serum PSA, discriminates between PCa patients and healthy individuals or patients with prostatitis or BPH. Plasma hTERT mRNA shows higher sensitivity (85% vs 83%), specificity (90% vs 47%), PPV (83% vs 56%), NPV (92% vs 77%) and AUC ROC (0.932 vs 0.651) than PSA with respect to PCa diagnosis. Pairwise comparison of hTERT mRNA and PSA ROC curves showed significant differences (p<0.001).

ROC curves and the best cut-off value were used to determine the sensitivity, specificity, positive predictive value (PPV) and negative predictive value (NPV) of each assay. The sensitivity and specificity of the plasma hTERT mRNA assay with respect to detecting PCa were 91% and 85%, respectively (best cut-off value 0.45), (best cut-off value 0.45, with an AUC ROC curve of 0.932±0.027), whereas the PSA assay showed a sensitivity of 83% and a specificity of 47% (best cut-off value 7.2 ng/ml, (best cut-off value 7.2 ng/ml, with an AUC ROC of 0.651±0.054 (Figure 3). The pairwise comparison of hTERT mRNA and PSA ROC curves confirmed significant differences (p<0.001) (Figure 3). PPV and NPV were higher for the hTERT mRNA assay than for the PSA assay. Positive and negative LR were 5.99 and 0.10, respectively, for the hTERT test and 1.57 and 0.37, respectively, for the PSA assay (Table 2). To further test the diagnostic accuracy of both tests in PCa diagnosis, we chose a high sensitivity (98%) for both tests and calculated the specificity and associated likelihood ratio. At this sensitivity, the plasma hTERT assay showed higher specificity (78% vs. 10%) and higher positive likelihood ratio (4.44 vs. 1.10) than the PSA test.

**Table 2:** Diagnostic accuracy of hTERT mRNA in comparison with a preoperative serum PSA test.

Diagnostic test	Cut-off point	Sensitivity (95%CI)	Specificity (95%CI)	PPV	NPV	LR +	LR −
hTERT mRNA	0.45	91.3% (79.2–97.5)	84.7% (73.0–92.8)	83.0%	92.2%	5.99	0.10
PSA(ng/ml)	7.2	82.6% (68.6–92.2)	47.5% (34.3–60.9)	56.3%	77.0%	1.57	0.37
hTERT/PSA combination	hTERT (0.45) PSA (7.2)	97.8% (86.4–99.9)	42.4% (35.2–65.0)	57.0%	96.2%	1.69	0.05
PSA(ng/ml)	4.0	100% (92.2–100.0)	8.47 (2.8–18.7)	47.5%	100%	1.09	0.00
PSA(ng/ml)	2.5	100% (92.2–100)	5.08 (1.1–14.2)	46.6%	100%	1.05	0.00

PPV: Positive predictive value; NPV: Negative predictive value; CI: confidence interval; LR: Likelihood ratio.

Combination of plasma hTERT mRNA and serum PSA in diagnosis for PCa resulted in a sensitivity of 98%, a specificity of 42%, a PPV of 57%, an NPV of 96%, an LR+ of 1.69 and an LR− of 0.05 (Table 2).

Interestingly, 5 of the 47 prostatitis patients that developed PCa over a five-year period showed higher mean hTERT mRNA values at diagnosis than the rest of the prostatitis group (1.08±0.80 vs. 0.45±1.00), although differences were not statistically significant (p = 0.26), probably due to the small number of cases analyzed.

### Plasma hTERT mRNA is Associated with Poor Prognosis Tumor Clinicopathological Parameters

Univariate analysis showed that plasma hTERT mRNA levels were significantly associated with poor prognosis tumor clinicopathological parameters such as Gleason score (p = 0.01), percent biopsy core positivity (p = 0.002), perineural invasion (p = 0.002), lymphovascular invasion (p<0.001) and clinical stage (p = 0.004). No statistically significant association was found between preoperative serum PSA levels and Gleason score (p = 0.10), percent biopsy core positivity (p = 0.34), perineural invasion (p = 0.35) or vascular/lymphatic invasion (p = 0.15). Nevertheless, significantly higher PSA values were observed in patients at an advanced clinical stage (p<0.01) (Table 3).

**Table 3:** Differences between hTERT mRNA and preoperative serum PSA levels according to prostate biopsy characteristics and final stage (adding clinical staging).

	N (%)	hTERT mRNA*	PSA (ng/ml)*
**Gleason score**			
6 (3+3)	21 (46.7)	0.8 (0.5–2.2) (p = 0.010)	8.7 (6.5–10.5) (p = 0.10)
7 (4+3)	16 (34.8)	2.4 (1.2–7.8)	10.5 (8.7–12.7)
>7	9 (18.5)	3.1 (1.4–9.1)	13.2 (8.7–34.1)
**Positive Biopsy Cores (%)**			
<50%	34 (74)	1.4 (0.6–2.7) (p = 0.002)	9.5 (7.5–13.2) (p = 0.34)
≥50%	12 (26)	3.7 (2.7–9.1)	10.7 (8.1–21.8)
**Perineural invasion**			
Yes	26 (56.5)	3.9 (2.4–9.1) (p = 0.002)	11.0 (9.5–26.4)(p = 0.35)
No	20 (43.5)	0.7 (0.5–1.4)	8.7 (7.4–10.6)
**Lymphovascular invasion**			
Yes	17 (37)	4.8 (2.8–9.1) (p<0.001)	10.9 (8.5–25.4) (p = 0.15)
No	29 (63)	1.0 (0.5–1.8)	8.8 (7.5–11.2)
**Stage**			
Organ-confined	33 (71.7)	1.2 (0.6–2.4) (p = 0.004)	8.7 (7.4–10.5)(p<0.01)
Locally-advanced	8 (17.4)	3.4 (1.6–7.6)	19.8 (13.4–30.3)
Metastatic	5 (10.9)	9.1 (4.4–12.9)	43.8 (11.6–88.4)

* Non-parametric Mann-Whitney U and Kruskal-Wallis tests were used for comparison of two medians and more than two medians, respectively.

Table 4 shows the results of the logistic regression analysis of pretreatment predictors of PCa: age (p = 0.04) and plasma hTERT mRNA (p<0.0001) were significant independent predictors of PCa, while serum PSA at diagnosis (p = 0.94), DRE (p = 0.43) and TRUS (p = 0.85) were not independent predictors.

**Table 4:** Results of the logistic regression analysis predicting PCa diagnosis.

Variable	Odds Ratio (95% CI)	p-value*
Age	0.89 (0.80–0.99)	0.04
Serum PSA at diagnosis	0.99 (0.87–1.14)	0.94
Plasma hTERT mRNA	11.07 (3.37–36.37)	0.0001
DRE	3.34 (0.16–68.28)	0.43
TRUS	0.78 (0.06–9.44)	0.85

DRE: Digital Rectal Examination; TRUS: Transrectal Ultrasonography; CI: Confidence Intervals;

* Logistic regression analysis, p–values less than 0.05 were considered statistically significant.

### Plasma hTERT mRNA is a Prognostic Factor of Biochemical Recurrence

**Figure 4:**
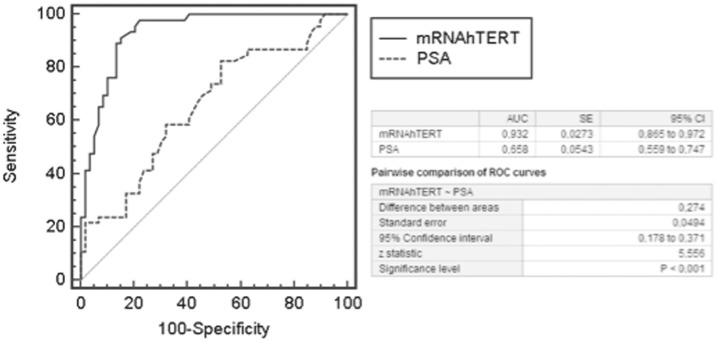
Efficacy of plasma hTERT mRNA and serum PSA as predictors of biochemical recurrence. ROC curves show that plasma hTERT mRNA, but not serum PSA, is a significant predictor of biochemical recurrence. Plasma hTERT mRNA shows higher specificity (91.8% vs 87.7%) and AUC ROC curve (0.932 vs 0.658) than PSA with respect to biochemical recurrence. The PSA assay shows higher sensitivity (71.4% vs 100%) than hTERT mRNA in predicting biochemical recurrence. Pairwise comparison of hTERT mRNA and PSA ROC curves showed significant differences (p<0.001).

**Figure 5:**
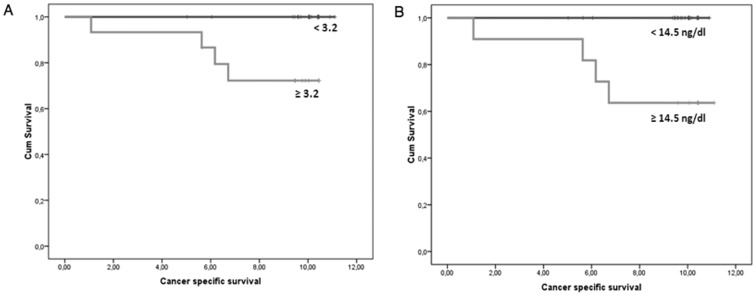
Kaplan-Meier analysis of time to biochemical recurrence. Kaplan-Meier curves indicate that there are significant differences in recurrence-free survival between high and low hTERT groups (A) but not between high and low serum PSA groups (B).

Plasma hTERT mRNA was shown to be a significant factor of biochemical recurrence. The plasma hTERT mRNA cut-off value for optimizing sensitivity and specificity in predicting biochemical recurrence was 3.15. At this cut-off value, sensitivity and specificity were 71.4% and 91.8%, respectively and the AUC ROC curve was 0.932±0.02 (Figure 4). Figure 5A shows the Kaplan-Meier curves for the two groups defined by the hTERT cut-off value (3.15). The recurrence-free survival time was significantly higher (p = 0.004; Chi-square test) in patients with hTERT mRNA <3.15 (mean±SD; 10.44±0.26 years; 95% CI: 9.92–10.96) than in patients with values ≥3.15 (mean±SD; 8.28±0.60 years; 95% CI: 7.10–9.41). On the other hand, preoperative serum PSA is not a predictor of biochemical recurrence. The optimal cut-off value for predicting biochemical recurrence was 14.5. Sensitivity and specificity were 100% and 87.7%, respectively and the AUC ROC curve was 0.658±0.05 (Figure 4). Kaplan-Meier curves for the two groups defined by the PSA cut-off value are shown in Figure 5B. No differences in recurrence-free survival (p = 0.38) were observed among patients with PSA <14.5 (mean±SD; 10.00±0.34 years; 95% CI: 9.34–10.68) and patients with PSA values ≥14.5 (mean±SD; 9.09±0.68 years; 95% CI: 7.77–10.42). Pairwise comparison of ROC curves showed significant differences (p<0.001) (Figure 4).

Predictors of biochemical recurrence were analyzed with COX regression analysis (Table 5). Multivariate analysis showed that hTERT mRNA (OR: 1.55; 95% CI: 0.99–2.43; p = 0.04) and stage (OR: 0.13; 95% CI: 0.01–1.30; p = 0.08) are significantly associated with biochemical recurrence, whereas none of the other analyzed factors showed significant association with biochemical recurrence.

**Table 5:** ORs and 95% CI for biochemical recurrence associated with prognosis factors.

Variable	Odds Ratio (95% CI)	p-value*
Plasma hTERT mRNA (cut-off: 3.2)	1.55 (0.99–2.43)	0.04
Serum PSA (cut-off: 14.5)	0.97 (0.88–1.07)	0.59
Gleason score	0.52 (0.20–1.33)	0.17
Age	0.96 (0.82–1.12)	0.59
Positive biopsy core (%)	1.06 (0.99–1.14)	0.11
Lymphovascular invasion	6.26 (0.212–185.15)	0.29
Perineural invasion	0.81 (0.02–28.79)	0.91
Treatment	0.15 (0.002–13.99)	0.41
Stage	0.13 (0.01–1.30)	0.08

CI: Confidence Intervals;

* COX regression analysis, p-values less than 0.05 were considered statistically significant.

## Discussion

Serum PSA has been widely used over the last decade in the diagnosis and follow-up of PCa. The major problems posed by this approach is the lack of cancer specificity and low predictive value of PSA, which result in an unnecessary number of prostate biopsies and/or failure to diagnose the disease in men with PSA levels below the cut-off value. Although high PSA levels are often associated with more aggressive tumors, PSA does not always correlate with the biological behavior of the disease, which leads to overtreatment and its consequences (increased cost, side-effects and patient anxiety). The challenge for clinicians is to distinguish between these different conditions and to avoid basing decisions purely on PSA measurements. As a consequence, new diagnostic tools are required to detect PCa as early and as accurately as possible in order to reduce the number of biopsies while successfully detecting as many cases of PCa as possible.

In this study, 105 patients with PSA values above 4.0 ng/ml underwent a prostate biopsy to rule out the presence of PCa. Only 46 of these patients (44%) were eventually diagnosed with PCa, whereas 47 (45%) and 12 (11%) were diagnosed with prostatitis and BPH, respectively. These results show that PCa screening based on PSA as the only indicator for a prostate biopsy lacks specificity and leads to a large number (59/105; 56%) of unnecessary interventions. Although different strategies have been developed in an attempt to increase the specificity of PSA, including measuring PSA velocity, levels of free/total PSA ratio, and the use of cut-off values specific to patient age and ethnic group, their clinical reliability has not been confirmed [2], [19]. Our group has previously demonstrated, in a small number of patients, that the quantification of plasma hTERT mRNA is a considerably sensitive and specific method for identifying PCa and that, when used in combination with serum PSA, is an effective method of PCa diagnosis [16]. Moreover, in a recent report we have shown that the plasma hTERT mRNA discriminates between clinically localized and locally advanced disease and is a predictor of recurrence in PCa patients [20]. We have now assessed the accuracy of plasma hTERT mRNA as a diagnostic tool for PCa in a larger number of patients and have explored its association with the clinicopathological characteristics of tumors. We have also explored the utility of plasma hTERT mRNA as a prognosis factor of biochemical recurrence. The results confirm and extend those of a previous report, as they indicate that plasma hTERT mRNA is a non-invasive tumor marker which can be used for PCa diagnosis, showing higher sensitivity (85% vs. 83%), specificity (90% vs. 47%), PPV (83% vs. 56%), NPV (92% vs. 77%) and LR ratios than the PSA assay. indicating that the plasma hTERT mRNA assay has a high discriminatory power and, therefore high potential utility as diagnostic test in PCa diagnosis. Some medical associations recommend reducing the PSA cut-off point from 4.0 ng/ml to 2.5 ng/ml in order to increase its sensitivity and therefore detect more cancers at an earlier stage. To further explore the diagnostic accuracy of the PSA assay, we chose two PSA values currently used in clinical practice (PSA ≥4.0 ng/ml and ≥2.5 ng/ml) and calculated their sensitivity, specificity, PPV and NPV and positive and negative LR. We have observed how reducing the PSA cut-off point increases sensitivity but reduces specificity and PPV. At these cut-off points for PSA, the plasma hTERT assay shows much higher specificity (85% vs. 8% and 5%), PPV (83% vs. 47% and 46%) and positive LR (5.99 vs. 1.09 and 1.05), but similar sensitivity (91% vs. 100%), NPV (92% vs. 100%) and negative LR (0.10 vs 0.00) (Table 3). Combination of plasma hTERT mRNA and PSA does not improve diagnostic accuracy. A limited increase in sensitivity (91% for hTERT vs. 98% for the hTERT/PSA combination) results in considerable reduction in specificity (85% vs. 42%), PPV (83% vs. 57%) and LR +(5.99 vs. 1.69).

Multivariate analysis identified age and plasma hTERT mRNA at diagnosis as significant independent predictors of PCa, whereas serum PSA at diagnosis, DRE and TRUS were not predictors in this regard.

Taken together, these results indicate that the plasma hTERT mRNA assay has a high discriminatory power and is, therefore, potentially useful for diagnosing PCa.

Interestingly, 5 of the 47 prostatitis patients that developed PCa over a five-year period showed higher mean hTERT mRNA values than the rest of their group. This finding provides further support for the idea that plasma hTERT mRNA has implications for the follow-up and minimal residual detection of the disease, although the number of patients in our study was too small to draw firm conclusions. Other authors have published similar results, reporting that telomerase RNA could be useful for diagnosis of advanced PCa and monitoring patients for detecting minimal residual disease [15]. It should be pointed out that hTERT RNA has been evaluated from plasma samples, thus transcription of hTERT from non-tumor sources, such as activated lymphocytes, could not be excluded. Moreover, the presence of plasma hTERT mRNA above the cut-off level may indicate the presence of circulating tumor cells, further supporting the idea that quantitative analysis of free-circulating RNA could be useful for diagnosing and monitoring PCa patients and may have implications in minimal residual disease detection.

The mechanism by which cell-free circulating RNA is released into the bloodstream is unknown, as is the source of circulating RNA. A hematopoietic origin of circulating RNA has been suggested, although the correlation in mutations, microsatellite alterations, promoter methylation in paired tumor and plasma/serum samples suggests that circulating RNA may originate from tumor tissue [21]. Higher cell-free circulating RNA levels have been found in patients with malignant lesions than in patients without tumors. However, high levels of circulating RNA are not cancer-specific and increased levels have been found in patients with inflammatory processes and benign lesions. Recent reports have correlated the levels of several tumor markers in plasma/serum with clinical and pathological characteristics of the disease suggesting that cell-free circulating RNA may provide biomarkers for cancer diagnosis, follow-up of patients after surgery and monitoring of treatment efficacy [22].

One of the most important findings of the present study is the association between high plasma hTERT mRNA values and clinicopathological parameters of tumors. To the authors’ knowledge, this is the first report showing that plasma hTERT mRNA is significantly associated with poor prognosis PCa tumor characteristics, such as Gleason score (p = 0.01), tumor stage (p = 0.004), and vascular (p<0.001) and perineural invasion (p<0.001).

Although the number of patients showing biochemical recurrence is limited (only seven patients), our results also indicate that plasma hTERT mRNA is a significant prognostic factor of recurrence. Kaplan-Meier survival curves confirmed the significant differences between groups so that patients with plasma hTERT mRNA levels above the cut-off value showed significantly diminished recurrence-free survival than patients with levels below the cut-off value. On the other hand, serum PSA is not a predictor of biochemical recurrence and no significant differences in recurrence-free time were observed between patients above and below the cut-off value. Multivariate analysis confirmed that plasma hTERT mRNA is (together with stage) an independent predictor of biochemical recurrence whereas none of the other analyzed factors (including PSA) showed significant association with biochemical recurrence.

Several authors have reported similar findings in other types of cancer, indicating that plasma hTERT mRNA may have prognostic implications [12]–[13], [23]–[24]. Miura et al. measured hTERT mRNA and Epidermal Growth Factor Receptor (EGFR) mRNA in serum from lung cancer patients and showed that hTERT mRNA was independently associated with tumor size, tumor number, presence of metastasis, recurrence and smoking. EGFR mRNA correlated with advanced clinical steps and both markers significantly decreased after surgery [12]. The same authors demonstrated that serum hTERT mRNA is useful for the diagnosis of gynecologic cancers. These patients show higher serum hTERT mRNA than those patients with benign diseases and healthy individuals. Serum hTERT mRNA independently correlated with clinical stage, CA125 and histological parameters in ovarian cancer [22]. Terrin et al. showed that plasma hTERT mRNA is a useful marker for detection and monitoring of colorectal carcinoma [17].

Besides telomerase activity, human cancer cells can also use a telomerase-independent mechanism called alternative lengthening of telomeres (ALT). Although the ALT mechanism is relatively common in sarcomas and astrocytomas, it has never been reported in PCa. Heaphy et al. have assessed 1.176 PCa tissues (including both adenocarcinomas and small cell carcinomas) and did not find a single ALT-positive tumor [25] indicating that the ALT mechanism is not used by PCa cells to maintain their telomeres. Consequently, the plasma hTERT mRNA assay should detect all PCa cases.

Several new biomarkers present in biological fluids are currently under evaluation to determine their potential in the earlier detection and prognosis of PCa. Altimari et al. showed that circulating free plasma hTERT DNA is higher in patients with PCa than in control subjects and correlates with tumor stage. The authors conclude that free plasma hTERT DNA is a promising biomarker for early diagnosis and monitoring of PCa [26]. Human glandular kallikrein 2 (hK2) has been reported to increase the precision of PSA-based prediction in patients with PSA levels below the standard cut-off thresholds for biopsy, and might be useful in determining clinically insignificant PCa. Prostate Cancer Antigen 3 (PCA3) is employed to measure PSA mRNA following a digital rectal exam. The PCA3 to PSA mRNA ratio (the PCA3 score) is calculated. The higher the PCA3 score the higher likelihood of PCa. PCA3 is reported to have a sensitivity of 69% and specificity of 79%. Two other serum biomarkers under investigation are proPSA and benign PSA (BPSA). BPSA is found at higher levels in hyperplastic tissue from patients with BPH, whereas proPSA is present in PCa. Together, these biomolecules may help to distinguish PCa from benign conditions and thus reduce the number of unnecessary biopsies [27]–[29]. Since plasma hTERT mRNA seems to have diagnostic and prognostic implications in PCa, it should be included in this list of markers in order to improve diagnostic accuracy and to help identify low-risk patients with more aggressive tumors before they develop into advanced or metastatic disease.

Considered as a whole, these biomarkers show promising results, although large-scale studies are necessary to adequately define their role in PCa screening and management.

Although the findings of the study are consistent, it presents some limitations that we would like to point out. Firstly, the number of patients showing biochemical recurrence [7] is relatively small to draw definitive conclusions about the usefulness of plasma hTERT mRNA as a prognostic factor of recurrence. Secondly, the patients were attended in only one Hospital (HCUV), which could introduce technical differences, such as sample collection procedures, transportation time from blood collection point to the laboratory, etc. that could modify the results. Therefore, further studies including a higher number of patients attended at several hospitals are required to validate the diagnostic and prognostic value of the hTERT mRNA assay.

Overall, the present results show that plasma hTERT mRNA is a useful non-invasive tumor marker for the molecular diagnosis of PCa, affording a greater diagnostic accuracy than the PSA assay. Furthermore, our results show that plasma hTERT mRNA is associated with poor prognosis tumor characteristics, and is a prognostic factor of recurrence at the molecular level, which may be of relevance to the follow-up and minimal residual detection of the disease.
